# Modelling Self-Organization in Complex Networks Via a Brain-Inspired Network Automata Theory Improves Link Reliability in Protein Interactomes

**DOI:** 10.1038/s41598-018-33576-8

**Published:** 2018-10-25

**Authors:** Carlo Vittorio Cannistraci

**Affiliations:** 1 Biomedical Cybernetics Group, Biotechnology Center (BIOTEC), Center for Molecular and Cellular Bioengineering (CMCB), Center for Systems Biology Dresden (CSBD), Department of Physics, Technische Universität Dresden, Tatzberg 47/49, 01307 Dresden, Germany; 2 Brain bio-inspired computing (BBC) lab, IRCCS Centro Neurolesi “Bonino Pulejo”, Messina, Italy

## Abstract

Protein interactomes are epitomes of incomplete and noisy networks. Methods for assessing link-reliability using exclusively topology are valuable in network biology, and their investigation facilitates the general understanding of topological mechanisms and models to draw and correct complex network connectivity. Here, I revise and extend the local-community-paradigm (LCP). Initially detected in brain-network topological self-organization and afterward generalized to any complex network, the LCP is a theory to model local-topology-dependent link-growth in complex networks using network automata. Four novel LCP-models are compared versus baseline local-topology-models. It emerges that the reliability of an interaction between two proteins is higher: (i) if their common neighbours are isolated in a complex (local-community) that has low tendency to interact with other external proteins; (ii) if they have a low propensity to link with other proteins external to the local-community. These two rules are mathematically combined in C1*: a proposed mechanistic model that, in fact, outperforms the others. This theoretical study elucidates basic topological rules behind self-organization principia of protein interactomes and offers the conceptual basis to extend this theory to any class of complex networks. The link-reliability improvement, based on the mere topology, can impact many applied domains such as systems biology and network medicine.

## Introduction

The problem of presence of noise in the topology of complex networks is of primary relevance in network and systems biology. A protein-protein interaction network (PPIN), a.k.a. protein interactome, can be modelled as an undirected graph: vertices represent proteins and edges represent interactions between proteins. PPINs are a paradigmatic example of noisy and incomplete networks and their analysis can be useful to enlighten and understand this problem also from a more general perspective in network science. In case of PPINs the existence of noise in the topology of the network is due to the presence of false-positive (FP) interactions detected during the experiments. In fact, despite the recent advancements, the FP rate of currently widely used experimental technologies is still high^1,2^. The development of methods that are able to assess the reliability of interactions is fundamental for denoising PPINs^3,4^, which is a pre-processing step that can be very beneficial in many network biology applications and tools, such as to name a few: (i) computational prediction of protein complexes from PPINs^5,6^; (ii) topological prediction of PPIs^7–9^; (iii) network-based inference of disease-related functional modules and pathways^10–12^; (iv) computational methods for constructing network-based ontologies of gene function, an example of which is the recently developed network-extracted ontology (NeXO)^13^.

**Figure 1:**
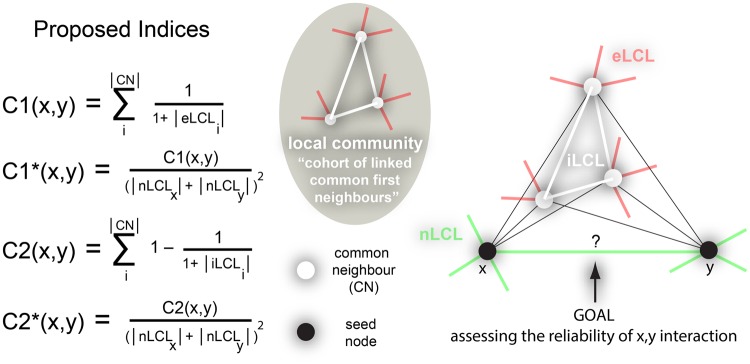
Extended paradigm on local community links. On the left, the mathematical formulae of the four novel local-community-based neighbourhood indices. On the right, an example considering one local community and the proposed subdivision of the seed node’s neighbourhood into: internal local-community-links (iLCL, white lines), external local-community-links (eLCL, red lines) and non local-community-links (nLCL, green lines).

Many computational approaches have been proposed to denoise and to assess the reliability of PPIs^3,4^, and among them topological approaches represent a new exciting field^3,4^. Topological denoising (or topological link reliability) methods are useful techniques that rely exclusively on the structural information (the binary graph adjacency matrix) provided by the PPIN topology, in order to assess a reliability score for each of the links (protein interactions) present in the network. The higher the score, the more likely the two proteins interact with each other. In practice, these approaches are equivalent to anomaly detection methods in machine learning: they take as input the network topology (for example, an adjacency matrix or a list of interactions) and they give as output a ranking of the network links in decreasing order of reliability. Topological reliability approaches are very versatile. They can be easily and rapidly applied as pre-processing steps^3,4^ to each of the aforementioned biological applications and tools because they do not require information other than the network structure. In addition, they are particularly convenient when the available biological information on the proteins being tested for interaction (seed proteins, see Fig. 1) is incomplete and unreliable, or when in general the prior biological knowledge is absent.

*Neighbourhood* methods are between the most used algorithms for topological link reliability of PPINs^8^. They are named so because they assign a score to any network interaction by relying on the topological properties of its seed proteins’ neighbourhood. They are local-structure similarity-based algorithms, hence they are simple, efficient and can deal with large-scale networks in a reasonable time^8^. More precisely, three algorithms are the points of reference among the neighbourhood algorithms used in network biology: Czekanowski-Dice (CD)^14^, Functional Similarity Weight (FSW)^15^, Adjusted-CD (ACD)^5,6^. Originally proposed to predict protein functions, they perform equally well for assessing the reliability of protein interactions, as was later demonstrated^5^.

On the contrary, global algorithms are named so because they assign a score by relying on the properties of the overall network topology at once, and the machine learning methods - such as the ones based on dimensionality reduction of the network topology in a geometrical space^1,7^ or the ones based on the clustering of the network in submodules^3^ – are between the most important. In particular, CAPPIC (Cluster-based Assessment of Protein-Protein Interaction Confidence), which is a machine learning method based on Markov clustering, was proved to be between the most effective^3^.

This article is dedicated and only focused to local-structure similarity-based algorithms and stems from two main observations. First: classical neighbourhood methods, such as common neighbours^16^, preferential attachment^16^, Adamic-Adar^17^, resource allocation^18^, etc., which are well-recognized in the field of network science, have never been tested in network biology for assessing link reliability. And second: previous studies on network reliability concentrated principally on yeast networks, testing the performance of the algorithms generally only on a reduced number of networks.

Here, together with CD, FSW, ACD and Interaction Generality (IG1)^19^ that are well-known in network biology, I test the network reliability performance of other 12 neighbourhood algorithms derived mainly from other fields of network science, such as link prediction and social networks. In addition, I propose 4 new algorithms and test and compare their performance in 10 different high-quality networks from 5 different organisms: 3 for yeast (S. cerevisiae), 1 for plant (A. thaliana), 1 for worm (C. elegans), 1 for fly (D. melanogaster), 4 for human (H. sapiens). In particular, I included one human network that is high-quality and structurally resolved^20^ (only those interactions in which the interacting domains of both partners, or their homologs, can be found in a 3D structure of an interaction were kept) in order to gauge the validity of a new proposed Gene-Ontology-based evaluation framework (for the evaluation of the network reliability algorithms) that is more conservative and restrictive than the one adopted in the past studies. Nevertheless, I would like to clarify that the aim of this study is not to scrutinize how reliable are the recent PPIN data released by large scale databases. Indeed, I have another objective, which is to investigate and discuss the different mechanistic rules of self-organization that characterize the reliability of the topology of PPIN regardless of the organism considered or of the period/year (I considered networks released in a period that covers 11 years: 2005 to 2015) in which they were combined and published.

Finally, the conceptual and mathematical formalizations of the LCP-theory and epitopological learning that I gave in my previous publication^8^ were quite immature. In this article, I dedicate an entire section to explain the fundaments and definitions of the LCP-theory. The section starts with a deep dive in the brain architecture, where the LCP-theory was initially developed, and progresses towards the generalization of the LCP-theory for any complex network and its interpretation in case of molecular networks. In addition, I revise and extend the LCP-theory considering that the local isolation of the operational units in the different local communities is important to carve the LCP architecture in the network, and this is guaranteed by the fact that the common neighbours minimize their interactions external to the local community. This is a topological property that I will demonstrate of particular importance for the architecture of protein interactomes.

## Methods

### Network datasets

In this study, nine different high-quality network datasets were considered. Each network was curated removing small isolated components of few nodes (because they present a poor topological connectivity and cannot be used in topological link reliability) following the same procedure suggested in previous publications^1,7,8,15,19,21–26^. The networks, along with their original number of nodes and edges are reported below.

Network 1: yeast (*S. cerevisiae*)^27^, comprises 4036 proteins and 10411 interactions.

Network 2: yeast (*S. cerevisiae*)^23^, comprises 4385 proteins and 12234 interactions.

Network 3: yeast (*S. cerevisiae*)^28^, comprises 3518 proteins and 9760 interactions.

Network 4: plant (*A. thaliana*)^29^, comprises 4519 proteins and 10721 interactions.

Network 5: worm (*C. elegans*) (BioGRID 3.1.92)^30^, comprises 2654 proteins and 4485 interactions.

Network 6: fly (*D. melanogaster*) (BioGRID 3.1.92)^30^, comprises 7887 proteins and 34711 interactions.

Network 7: human (*H. sapiens*)^20^, comprises 1579 proteins and 3460 interactions. This is a high quality and structurally resolved network, i.e. the interfaces of its interactions were structurally resolved using a homology modelling approach^20^. The reliability of the interactions in this PPIN should be significantly higher than in the other networks.

Network 8: human (*H. sapiens*)^28^, comprises 7831 proteins and 24241 interactions. This network was both systematically and manually curated.

Network 9: human (*H. sapiens*)^31^, comprises 4100 proteins and 13358 interactions.

Network 10: human (*H. sapiens*)^2^, comprises 13460 proteins and 141296 physical interactions experimentally documented in human cells, including protein-protein and regulatory interactions, metabolic pathway and kinase-substrate interactions, representing one of the largest and completed blueprints of the human interactome^2^.

The web-links for downloading these networks are provided in the respective articles related with the dataset publication. The adjacency matrices extracted for each of these networks and that were used for the analysis conducted in this article are available at the web-link provided in the Declaration section at the bottom of the article.

### Node neighbourhood based models

These algorithms assign a score to any network interaction by relying on the topological properties of the nodes that are neighbours of the seed proteins (Fig. 1). The common neighbours (CN) index^16^ is the predecessor of these algorithms and follows the natural intuition that the likelihood that two nodes x and y interact increases if their sets of first-node-neighbours N(x) and N(y) overlap substantially (the notation | | indicates the number of elements in the set):

$$CN(x,y)=|N(x)\cap N(y)|$$

The other node neighbourhood indices are often a variation or generalisation of CN. Adamic & Adar (AA)^17^ and Resource Allocation (RA)^18^ give more importance to common neighbours with low degree:

$$\begin{array}{ccc}AA(x,y) & = & \sum _{i}^{|CN|}\frac{1}{{\rm{l}}{\rm{o}}{\rm{g}}|N(i)|}\\ RA(x,y) & = & \sum _{i}^{|CN|}\frac{1}{|N(i)|}\end{array}$$

where |N(i)| is the degree of the node common neighbour *i*. Preferential Attachment (PA)^16^ is the degree product of nodes x and y:

$$PA(x,y)=|N(x)|\cdot |N(y)|$$

However, Cannistraci *et al*.^8^ recently gave an interpretation of PA in function of CN based on the fact that |N(x)| = |nLCL(x)| + CN(x):

$$PA(x,y)=|nLCL(x)|\cdot |nLCL(y)|+|nLCL(x)|\cdot CN(x,y)+|nLCL(y)|\cdot CN(x,y)+CN{(x,y)}^{2}$$

where nLCL(x) are the non local-community-links of node x: links of the seed node x that are not related with the local community (for details, see Fig. 1 and next section 2.3).

An important subclass of the node neighbourhood based indices is obtained by normalizing CN. Jaccard (JC)^32^, Sørensen-Czekanowski-Dice (SCD)^14,33,34^ and Leicht-Holme-Newman (LHN)^35^ are the landmarks normalised indices in complex networks:

$$\begin{array}{rcl}JC(x,y) & = & \frac{CN(x,y)}{|N(x)\cup N(y)|}\\ SCD(x,y) & = & \frac{2\cdot CN(x,y)}{|N(x)|+|N(y)|}\\ LHN(x,y) & = & \frac{CN(x,y)}{|N(x)|\cdot |N(y)|}\end{array}$$

For correctness, the Sørensen index (S) and Czekanowski-Dice dissimilarity (CD) are related according to the formula: S = 1 − CD. Here for simplicity I will use the formula of S, and I will name this index SCD in the reminder of the article.

To conclude, I consider also the Functional Similarity Weight (FSW)^15^ and the Adjusted-CD (ACD)^5,6^ that are more elaborated types of normalised indices, which adjust the normalisation in case the seed proteins have a lower number of interactions than the average node degree in the network. These are landmark algorithms in network biology and I invite the reader to refer to the original publications for more details. Here, they are used as reference, but it is not in the scope of this article to discuss them, since the literature on this subject is exhaustive. As shown by Chen *et al*.^22^, FSW and SCD outperform methods such as IG1^19^, IG2^25^ and the Interaction Reliability by Alternate Pathways (IRAP), which are outdated types of algorithms employed at the beginning in this research area (i.e. interaction reliability) of network biology. Given the fact that IG2 and IRAP are very computationally expensive^21^, I decided to adopt only IG1 (exclusively as a baseline that offers a lower-bound in the evaluations).

### Local-community-paradigm (LCP) based models

The node neighbourhood indices are focused on the common neighbours, therefore on a group of nodes and their proprieties. Cannistraci *et al*.^8^ proposed a strategic shift from nodes to links (in particular from nodes to community links, Fig. 1) that represents a new way to treat local-structure similarity-based measures in complex networks^8^. More specifically, a theory to model local-topology-dependent link-growth in complex networks called local-community-paradigm (LCP) was suggested^8^. The LCP-theory^8,36–39^ is a brain-network bioinspired theory, the origin of which will be carefully discussed in the next section. It holds that for modelling link prediction in complex networks, the information content related with the common neighbour nodes should be complemented with the topological information emerging from the interactions between them. The cohort of common neighbours and their cross-interactions form what is called a local community; the cross-interactions between CNs are called local community links (LCLs). In order to demonstrate the validity of the theory on several classes of networks, different classical node-based link prediction techniques like CN, JC, AA, RA and PA were reinterpreted according to the LCP-theory^8^, by introducing mathematical terms related with the LCLs in their formulae. Notably, the LCP-theory generated the pioneering design of a novel family of mechanistic parameter-free neighbourhood-based models not only specific to monopartite networks. In fact, recently the LCP-theory and the relative derived models have been proven valid also for bipartite complex networks^36^. The LCL is the first of these new topological-similarity measures that plays the part of a prediction model, and it is defined as the sum of the internal links between the common neighbours (Fig. 1). These links were simply defined as LCLs in previous publications^8,36^ and hereby are more specifically redefined as internal local community links (iLCL):

$$LCL(x,y)=\sum _{i}^{|CN|}\frac{|iLCL(i)|}{2}$$

Cannistraci-Alanis-Ravasi (CAR) is the local-community-link based correction of CN and is defined as:

$$CAR(x,y)=CN(x,y)\cdot LCL(x,y)$$

Cannistraci-Adamic-Adar (CAA) and Cannistraci-resource-allocation (CRA) are the local-community-link based corrections of AA and RA respectively, and are defined as:

$$\begin{array}{ccc}CAA(x,y) & = & \sum _{i}^{|CN|}\frac{|iLCL(i)|}{{{\rm{l}}{\rm{o}}{\rm{g}}}_{2}|N(i)|}\\ CRA(x,y) & = & \sum _{i}^{|CN|}\frac{|iLCL(i)|}{|N(i)|}\end{array}$$

Cannistraci-preferential-attachment (CPA) is the local-community-link based correction of PA and is defined as:

$$\begin{array}{lll}CPA(x,y) & = & |nLCL(x)|\cdot |nLCL(y)|+|nLCL(x)|\cdot CAR(x,y)+|nLCL(y)|\\  &  & \cdot CAR(x,y)+CAR{(x,y)}^{2}\end{array}$$

Cannistraci-Jaccard (CJC) is the local-community-link based correction of JC and is defined as:

$$CJC(x,y)=\frac{CAR(x,y)}{|N(x)\cup N(y)|}$$

### A deep dive in the brain architecture: towards the origin of the LCP-theory and the definition of local-ring network automata

In 1949, Donald Olding Hebb advanced a *local learning rule* in neuronal networks that can be summarized in the following: neurons that fire together wire together^40^. In practice, the Hebbian learning theory assumes that different engrams (memory traces) are memorised by the differing neurons’ cohorts that are co-activated within a given network. Yet, the concept of wiring together was not further specified, and could be interpreted in two different ways. The first interpretation is that the connectivity already present, between neurons that fire together, is reinforced; whereas, the second interpretation is the emergence and formation of new connectivity between non-interacting neurons already embedded in a interacting cohort.

The first interpretation has been demonstrated in several neuroscientific studies, where it was proven that certain forms of learning consist of synaptic modifications while the number of neurons remains basically unaltered^41–43^. A first mathematical model of this learning process was implemented in the Hopfield’s model of associative memory, where neuron-assemblies are shaped during engram formation by a re-tuning of the strengths of all the adjacent connections in the network^44^. It is important to specify that neuronal networks are over-simplified models and between two nodes (that represent two neurons) only one unique connection, which is deceptively called ‘synapsis’, is allowed. This unique artificial synapsis is a network link with a weight (or strength) and abstractly represents in a unique connectivity all the multitude of synapses that can occur between two real neurons in a brain tissue. For non-computational readers, I stress that the word ‘synapsis’ used in computational modelling of artificial neural networks might be misleading for neurobiologists, and should be intended as a mere link between two nodes of a network that comprehensively symbolizes the strength of all the real biological synapses connecting two neurons. Here, and in the reminder of this paragraph, I will refer only to this artificial neural network model where a link between two nodes (neurons) indicates an abstract interaction between them. In fact, although this artificial network model is based on evident simplifications, it demonstrated to be a powerful tool to simulate learning processes of intelligent systems^44,45^.

Surprisingly, the second possible interpretation of the Hebbian learning – a cohort of interacting neurons that fire together, give rise to new connections between non-interacting neurons in the cohort - to the best of my knowledge was never formalized as a general paradigm of learning, and therefore it was never employed with success to modify the architecture of abstract neural networks to simulate *pure topological learning*. I acknowledge the existence of studies that investigate how neuronal morphology predicts connectivity^46^. For instance, Peters’ rule predicts connectivity among neuron types based on the anatomical colocation of their axonal and dendritic arbors, providing a statistical summary of neural circuitry at mesoscopic resolution^46^. However, no paradigms were proposed to explain the extent to which new connections between non-interacting neurons could be predicted in function of their *likelihood* to be collectively co-activated (by firing together) on the already existing network architecture. This likelihood of localized functional interactions on the existing neural network can be influenced by external factors such as the temporal co-occurrence of the firing activity on a certain cohort of neurons, and by other factors that are intrinsic to the network architecture such as, among the most important, the *network topology*.

In 2013 Cannistraci *et al*. noticed that considering only the network topology, the second interpretation of the Hebbian learning could be formalized as a mere problem of topological link prediction in complex networks. The rationale is the following. The network topology plays a crucial role in isolating cohorts of neurons in functional communities that naturally and preferentially - by virtue of this predetermined local-community topological organization - can perform local processing. In practice, the local-community organization of the network topology creates a physical and structural ‘energy barrier’ that allows the neurons to preferentially fire together within a certain community and therefore to add links inside that community, implementing a type of local topological learning. In few words: the local-community organization influences (by increasing) the likelihood that a cohort of neurons fires together because they are confined in the same local community, consequently also the likelihood that they will create new connections inside the community is increased by the mere structure of the network topology. Inspired by this intuition, Cannistraci *et al*. called this local topological learning theory *epitopological learning*, which stems from the second interpretation of the Hebbian leaning. The definition was not clearly given in the first article^8^ that was quite immature, and therefore I now provide an elucidation of the concepts behind this theory by offering new definitions. *Epitopological learning* occurs when cohorts of neurons tend to be preferentially co-activated, because they are topologically restricted in a local community, and therefore they tend to facilitate learning by forming new connections instead of merely retuning the weights of existing connections. As a key intuition, Cannistraci *et al*. postulated also that the identification of this form of learning in neuronal networks was only a special case, hence the *epitopological learning* and the associated *local-community-paradigm (LCP)* were proposed as local rules of learning, organization and link-growth valid in general for topological link prediction in any complex network with LCP architecture^8^. On the basis of these ideas, they proposed a new class of link predictors that demonstrated - also in following studies of other authors - to outperform many state of the art local-based link predictors^8,47–53^ both in brain connectomes and in other types of complex networks (such as social, biological, economical, etc.). In addition, they proposed that the local-community-paradigm is a necessary paradigm of network organization to trigger epitopological learning in any type of complex network, and that LCP-correlation^8^ is a measure to quantitatively evaluate the extent to which a given complex network is organized according to the LCP. The LCP-correlation is generally computed as the Pearson correlation (but also other types of measures of association among variables can be used) between two variables whose size is equal to the number of links in the network. The first variable is the number of common neighbours (which create a local community) associated to each link of the network. The second variable is the number of local community links (connections among common neighbours) associated to each link of the network^8^. The LCP-correlation measures whether the number of interactions between the common neighbours is a function that increases with the number of common neighbours in the local community. Complex adaptive networks with weak-links that make local processing and global delivery generally follow the LCP organization (the LCP-correlation is generally ≥0.7), whereas the networks that do not follow the LCP organization (LCP-correlation ≤ 0.3) present strong-links, they are not clustered and they are suitable for storage or mere delivery of energy or information. It is very rare to find networks that have a LCP-correlation between 0.3 and 0.7^8^.

In conclusion, the LCP originated from the initial idea to explain how the network topology indirectly influences the process of learning a memory by adding new connections in a network of neurons, and consequently generalized to advocate mechanistic modelling of topological growth and self-organization in real monopartite^8^ and bipartite^36^ complex networks, with a significant impact also on prediction of drug-target interactions exploiting exclusively bipartite network topology^37^. This explains the title of the present article and clarifies the theoretical fundaments behind our results, which derive from molecular networks.

Protein interaction networks display a clear LCP architecture^8^, where protein complexes are confined in local and topologically isolated network structures, which are often coincident with functional network modules that play a crucial role in molecular circuits. The key generalized idea behind the LCP network architecture is that, for instance, a local community of neurons or proteins should take functional advantage of being confined in a local assembly of operational units. Each local assembly - if it is properly activated by an external signal coming from another region of the network - performs a functional operation by means of a structural remodelling of the internal connectivity between the operational units that are embedded in the network local community. The systems supported by LCP network architecture are very dynamic and react to a stimulus with a local plastic remodelling. In case of operational units such as neurons, the local community remodelling can implement for instance a learning process. In case of operational units such as proteins, the local community remodelling is instead necessary to implement for instance a biological process, which emerges by the molecular-complex rearrangement in the 3D space.

The previous conceptual and mathematical formalizations of the LCP-theory were immature and put more emphasis on the fact that the information content related with the common neighbour nodes should be complemented with the topological information emerging from the interactions between them. However, in this new study I would like to remark that the local isolation of the operational units in the different local communities is equally important to carve the LCP architecture in the network, and this is guaranteed by the fact that the common neighbours minimize their interactions external to the local community. This minimization forms in practice a sort of topological energy barrier, which in turn confines the signal processing to remain internally to the local community. In the next paragraph I will revise the LCP idea and its mathematical formalization in order to concretely take into account also the *minimization of the external links*. However, in this article I will discuss the implications of this theoretical revision only on modelling of protein interactomes, leaving to other studies the investigations of its impact on other types of networks. A recent study of Narula *et al*.^39^ shows that local-community-paradigm and epitopological learning can enhance our understanding of how local brain connectivity is able to process, learn and memorize chronic pain^39^. Besides, another recent study of Muscoloni *et al*.^38^ discusses how local parameter-free mechanistic models to predict link-growth in complex networks (such as the common neighbours and LCP-based indices discussed in the previous section) can be interpreted as network automata that compute the likelihood to close ‘local rings’ in the network whenever a link is missing in the topology. The *local ring* is the closure of a ‘local tunnel’ obtained by adding to the topology the missing link for which is computed the likelihood to appear. The *local tunnel* is the ensemble of all the local paths (which can be the smallest shortest-paths definable on a given network topology or the paths of a fixed arbitrary length that connect two nonadjacent nodes) which connect two nonadjacent nodes (extremities of the tunnel), and the common neighbours are all the nodes embedded in the tunnel structure, therefore they are an estimation of the size of the tunnel. For more details, please refer to the study of Muscoloni *et al*.^38^ that discusses also how some of these network automata models, such as the Cannistraci-resource-allocation (CRA), seem strongly related and able to predict the growth of network topology which is associated to hyperbolic geometry.

### New LCP-revised models

In this article, I revise and extend the paradigm on local community links (LCL). I propose to subdivide the neighbourhood of the seed nodes (Fig. 1) into: internal local-community-links (iLCL), external local-community-links (eLCL), and nonlocal-community-links (nLCL). iLCL are common neighbours’ links that interact only with other common neighbours in the local community. eLCL are common neighbours’ links that do not interact with the common neighbours and the seed proteins. nLCL are seed nodes’ links that do not interact with the local community of common neighbours. See Fig. 1 for a paradigmatic example which reports the case of one local community.

For correctness I clarify that in the previous publications^8,36^ I just generically defined and applied the name LCL to the same type of links that now, in the present article and according to the proposed extended definition, are relabelled with the new name of iLCL. I also make clear that in the past publications eLCL and nLCL were not identified and distinguished with respect to the other links present in the network. Furthermore, although in Fig. 1, for simplicity and clarity of the representation, only one local community is connected with the seed proteins, in a real scenario multiple disjointed local communities might be connected with the same seed nodes, but this multiple local-community-configuration would not affect the definition of iLCL, eLCL and nLCL I provided. Finally, a single common neighbour is considered a degenerate case of isolated local community with one node. According to these new definitions, I here propose four novel local-community-based parameter-free neighbourhood mechanistic models, which, as recently discussed in another article of mine^38^ (and briefly summarized in the previous section on the origin of the LCP theory), can be intended as local-ring network automata. Their mathematical formulae are given below together with an explanatory plot in Fig. 1. The first of these models is:

$$C1(x,y)=\sum _{i}^{|CN|}\frac{1}{1+|eLCL(i)|}$$

C1 is a mechanistic model based on a rule that can help to understand the importance to minimize links external to the local community – in the formula indicated by the term eLCL(i) – in order to predict the likelihood of a new link to occur or the reliability of an existing link between the seed nodes *x* and *y*. In the context of our study this model can help to disclose whether the reliability of an existing protein interaction is higher when the local community, although it is composed by many common neighbour proteins, remains isolated in a well-defined protein complex that minimizes its external interactions. The second model is:

$$C{1}^{\ast }(x,y)=\frac{C1(x,y)}{{(|nLCL(x)|+|nLCL(y)|)}^{2}}$$

C1* is a normalization of C1 that corrects the formula penalizing interactions between seed nodes that, although can have many common neighbours which are isolated in a local community, have also many nonlocal-community-links which in the formula are indicated by the terms nLCL(x) and nLCL(y) at the denominator. The rationale is that while a consistent number of common neighbours that are isolated (because they do not have external local-community-links, eLCL) indicates topological proximity of two seed nodes, on the other side if two seed nodes have a significant amount of nonlocal-community-links, nLCL, which overcome the number of common neighbours, this might indicate that the two seed nodes dominate far apart regions of the topology. In fact, the idea is that adjacent nodes with a high external degree (where the external degree is computed considering the number of neighbours not in common) should be geometrically far because they represent hubs without neighbours in common, which - according to the theory of navigability of complex networks presented by Boguñá *et al*.^54^ - tend to dominate geometrically distant regions. This same notion was also adopted with success in previous articles of mine: to formulate a dissimilarity kernel for network community detection by affinity propagation^55^; to propose the repulsive part of a pre-weighting rule, which was designed to approximate the geometrical distance between nodes in the topology, as first step to implement what we named the ‘coalescent embedding’ algorithm for mapping complex networks in the hyperbolic space^56^. A final intuitive remark (a speculation that I do not prove in this article, but it is emerging considering also the results gained in previous articles^8,36^ where the Jaccard index has bad link prediction performance) is that this normalization for the nLCL might play an important role in link-reliability because it relates to the evaluation of the adjacent links that are already in the network topology, while it can be less important and even harmful in designing a model for link-prediction. The reason is that the number of nonadjacent links considered for link prediction is in general (especially in large networks) much higher than the adjacent links which form the network topology. This implies that the combination of nonadjacent links that are close in the topology (because they are in the same local topological region) but that, just by chance, can have high number of external links is much higher than in the case of link reliability, causing as a matter of fact a wide and improper over-penalization of candidate links which otherwise would be highly ranked. This hypothesis needs to be further explored in future studies and might be considered a starting point to understand why the Jaccard index performs fairly in link-reliability but has low performance in link-prediction. The third model is:

$$C2(x,y)=\sum _{i}^{|CN|}1-\frac{1}{1+|iLCL(i)|}$$

C2 is a mechanistic model based on a rule that can help to understand the importance to maximize links internal to the local community – in the formula indicated by the term iLCL(i) – in order to predict the likelihood of a new link to occur or the reliability of an existing link between the seed nodes *x* and *y*. In the context of our study this model can help to disclose whether the reliability of an existing protein interaction is higher when the local community (which is composed by many common neighbour proteins) tends to establish many internal interactions regardless of the fact that, by minimizing the external interactions, it can form a well-defined protein complex. The fourth model is:

$$C{2}^{\ast }(x,y)=\frac{C2(x,y)}{{(|nLCL(x)|+|nLCL(y)|)}^{2}}$$

C2* is the normalized version of C2 according to the same rationale discussed above for C1*. To minimize redundancy in the article, further motivations for the rationale and the meaning underlying the mathematical formulae of these four new models are directly provided in the Results and Discussion section, where, with the help of the results gathered from the simulations in different networks, I intend to simplify the understanding of the topological ‘principia’ behind these mathematical formulae.

### New Gene-Ontology-based evaluation framework

Gene ontology (GO) is the gold standard (in the sense that it is the best available benchmark under reasonable conditions) employed in the past publications for the evaluation of the performance of algorithms for link reliability. The rationale adopted in the past studies is that PPIs that are involved in the same biological process (BP), have similar molecular function (MF) or are located in the same cellular component (CC), are very likely to occur^1,7,8,15,19,21–26^. All the past studies on methods for assessing link reliability adopted GO semantic similarity for determining the precision in reliability in each of the GO categories: MF, BP and CC. Below, I describe the procedure adopted in the previous studies.

**Figure 2:**
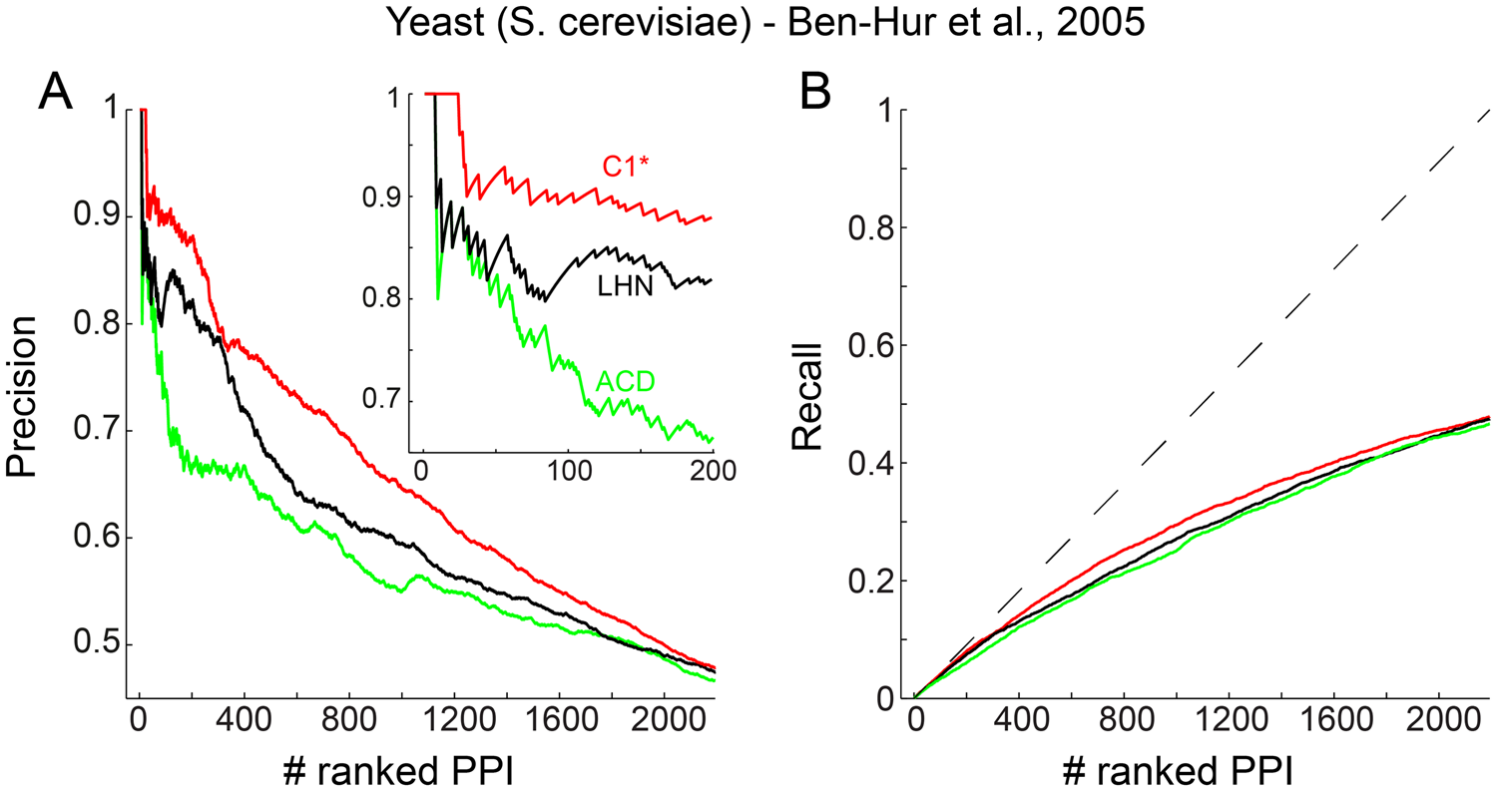
(**A)** Precision curves of the first-ranked methods (the first for each of the three considered classes) in network 1 considering the first 2000 ranked PPI. Inset on the upper-right of the figure reports the enlarged curves for the first 200 ranked interactions. (**B)** Recall curves of the same aforementioned methods. The dashed line indicates the best possible performance.

**Figure 3:**
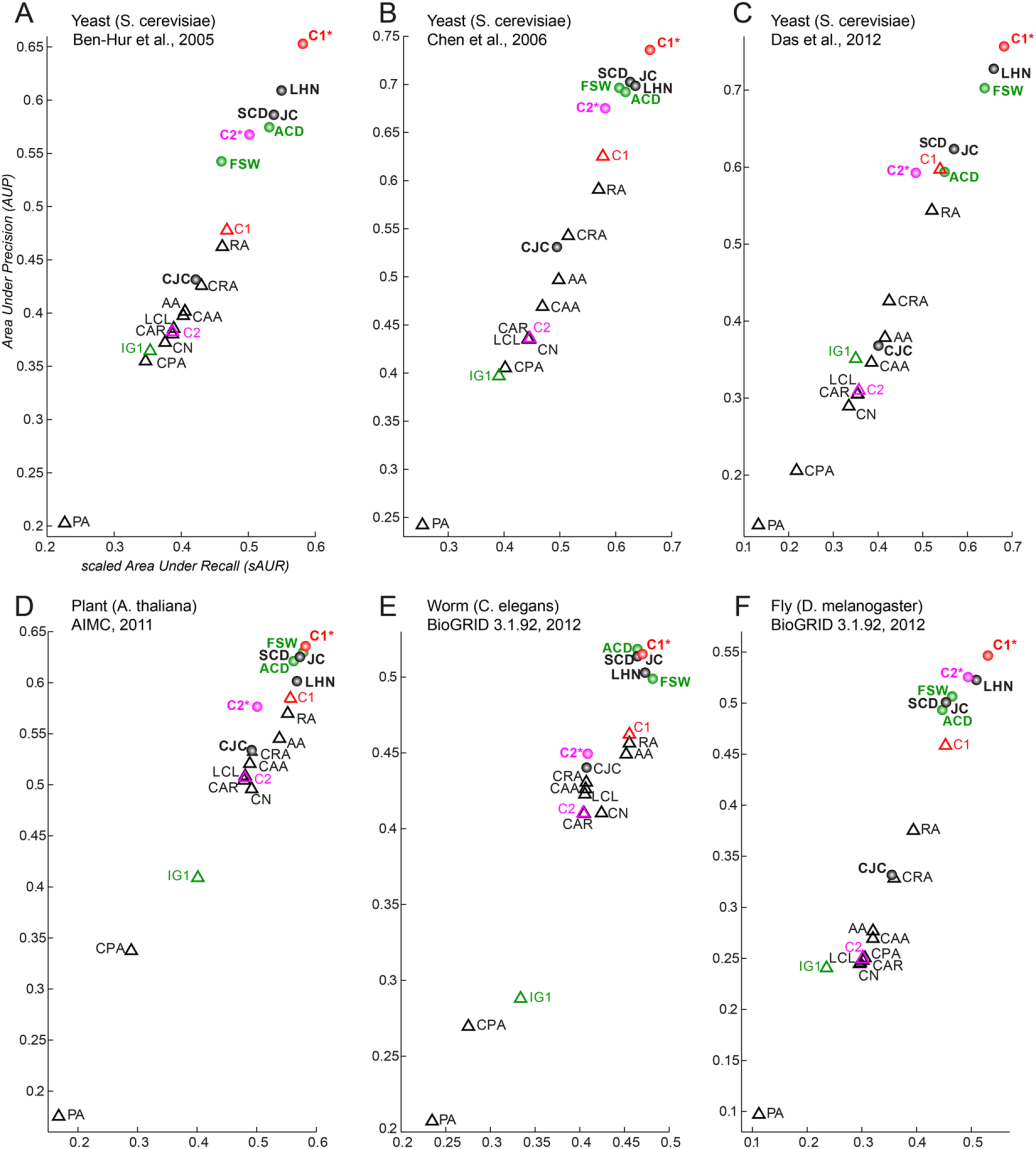
Performance evaluation in network 1, 2, 3, 4, 5, 6. Different colours are used to distinguish: the classical network science reliability models (black); the traditional network biology models (green); the two new local-community models based on the rule C1 and its normalized version C1* (red); the two new local-community models based on the rule C2 and its normalized version C2* (magenta). The triangle symbol is adopted to mark the non-normalised algorithms.

**Figure 4:**
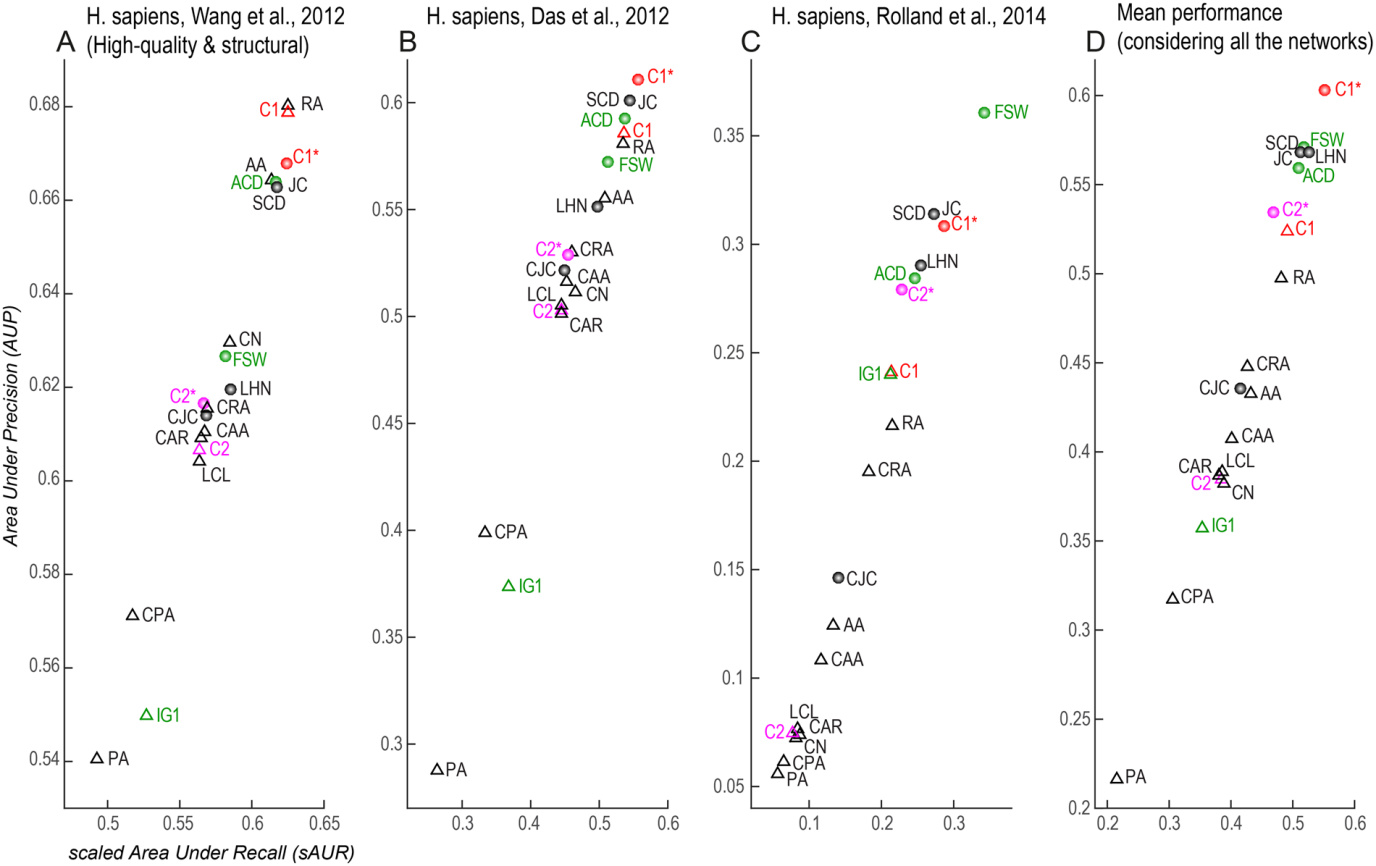
Performance evaluation in network 7, 8, 9. Different colours are used to distinguish: the classical network science reliability models (black); the traditional network biology models (green); the two new local-community models based on the rule C1 and its normalized version C1* (red); the two new local-community models based on the rule C2 and its normalized version C2* (magenta). The triangle symbol is adopted to mark the non-normalised algorithms. (**D)** Mean performance of each method computed as average sAUR (x-axis) and AUP (y-axis) on the nine considered networks.

**Figure 5:**
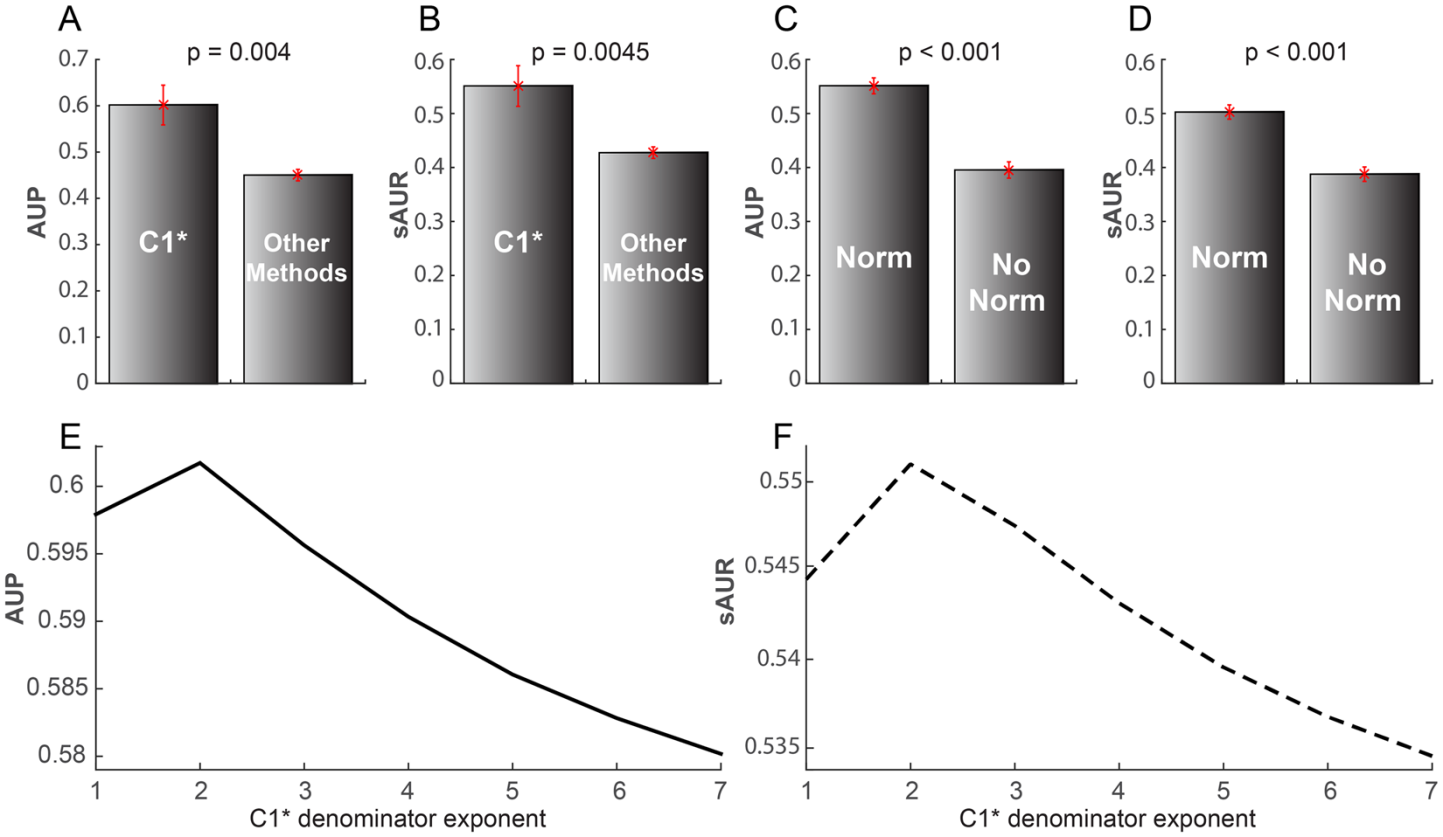
(**A,B**) Comparison between C1* mean performance and the mean performance of the other methods across all the networks according to AUP and sAUR. (**C,D)** Comparison between the mean performance of normalized and non-normalized methods across all the networks according to AUP and sAUR. The standard error is reported as a red colour deviation on top of each bar that indicates the mean value. P-values are reported on top of each panel, indicating the level of significance of the comparison. (**E,F)** mean AUP and sAUR values across all the networks computed for the C1* algorithm considering different denominator exponents.

**Figure 6:**
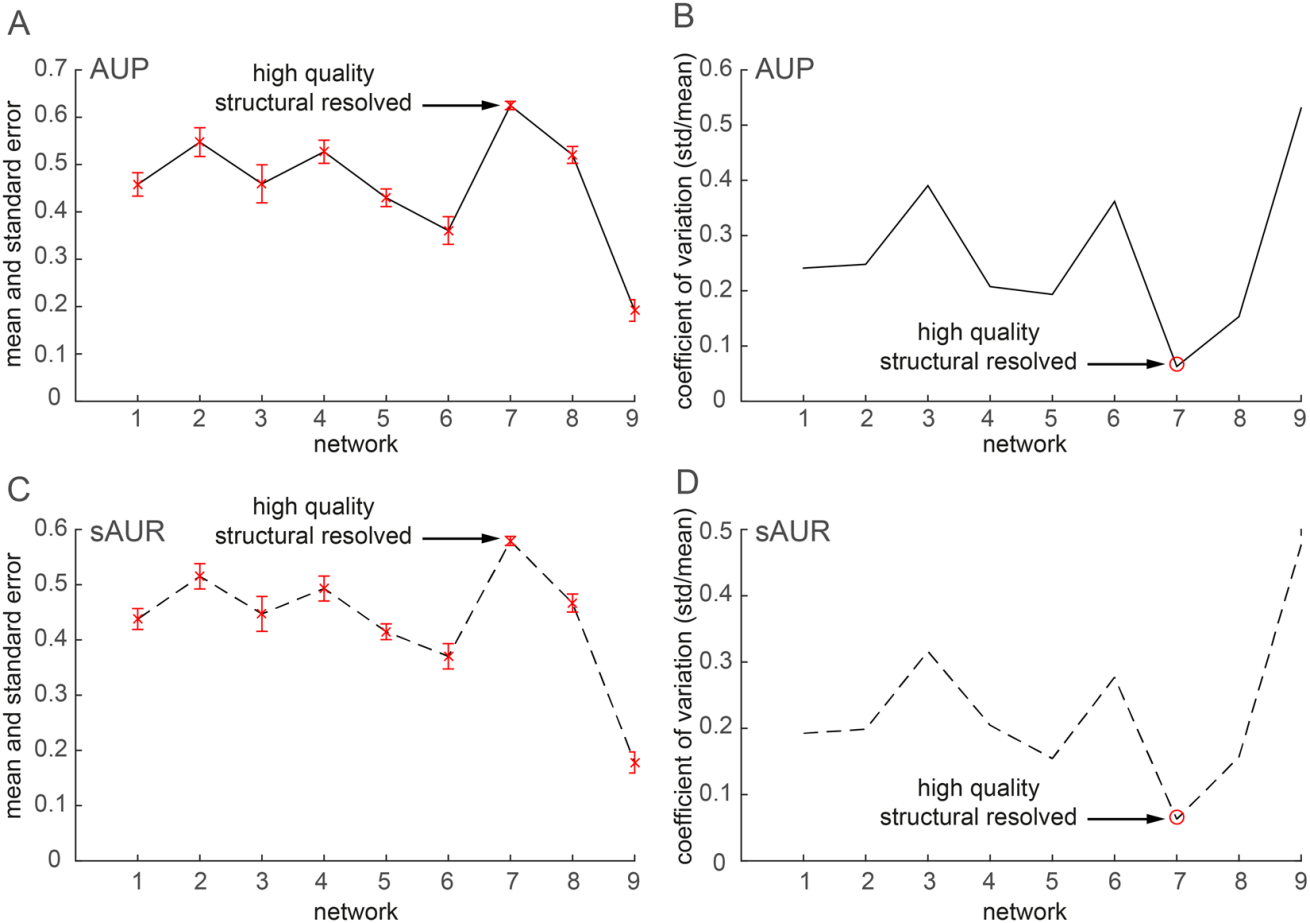
New GO-based evaluation framework. (**A**) Mean AUP values (y-axis, computed as average of all the methods for each network) and standard errors (red bars) for each network (x-axis). (**B)** Coefficient of variation (y-axis) of the AUP values for each network (x-axis). (**C)** Mean sAUR values and standard error for each network. (**D)** Coefficient of variation of the sAUR values for each network.

The similarity between GO terms is measured for all the pairs of proteins in the PPINs using the R package GOSemSim^57^ that implements the Wang GO semantic similarity method^58^. The GOSemSim function takes as input the list of proteins that form the PPIN, annotates them, computes the Wang GO semantic similarity between proteins and outputs an adjacency matrix whose entries are the GO similarities for every PPI. There are several GO semantic similarities^59^ that were originally developed for natural language taxonomies and it is not known if they are fully suitable for GO. Wang’s measure was designed specifically for the GO and its values range between 0 (if the two proteins do not have a similar MF, BP or CC) and 1 (if the proteins share one or more identical GO terms). Whenever the Wang similarity is in the high end of the range, the proteins being analysed can be considered analogous in their MF, BP, or CC^58^. Thus, as suggested in previous studies^1,7,8,15,19,21–26^ only those protein interactions with Wang similarity above 0.5 are considered reliable (‘true’) according to one of the GO categories. Finally, a precision curve is drawn for each of the three GO categories. For example: considering a sensitivity step of 10 interactions, the precision of the first 10 ranked interactions is evaluated creating a first point of the precision curve; then the precision of the first 20 ranked interactions is evaluated creating a second point of the precision curve; and so on until the end of the list of ranked interactions. Figure 2A reports an example of the precision curve (using sensitivity step 1) indicating the performance in network 1 of the first-ranked methods for each of the three topological-reliability classes: new proposed in this article (red colour), general-complex-network (black colour) and network biology (green colour). Generally, the reliability algorithms are evaluated comparing their performance separately in each of the three categories. The higher the profile of the precision curve in each of the GO categories, the better the algorithm performance. To quantitatively summarise the performance of the technique in one numeric value, the area under the precision curve (AUP) is adopted. In Fig. 2A, I adopt a new strategy that will be discussed below and that considers interactions as ‘true’ when they simultaneously share a similar BP and CC. In addition, I also estimate the area under the recall curve (AUR), which is an important evaluation generally neglected in previous studies. Since the AUR for the best predictor is equal to 0.5 (see triangle area under the dashed line in Fig. 2B), in order to scale the AUR values between 0 and 1 I decided to report in the Figs 3–6 the value sAUR = 2*AUR. Although AUP and AUR offer a more detailed view of the performance of each model, I also report the area under the precision-recall curve (AUPR) to compare their performance considering a trade-off between precision and recall capability. Therefore, the AUPR values for each GO annotation (BP, CC and MF) and for the new proposed evaluation strategy are reported. In contrast, the area under the receiver operating characteristic curve (AUC) has recently been proved to be deceptive in comparison to precision and recall^60^. AUC should be avoided in evaluation of link-prediction and link-reliability in complex networks because it puts emphasis also on the negative set of interactions that particularly in biology is not well-defined, indeed a missing PPI interaction is not a negative interaction.

Finally, it is important to mention that I am aware that the use of GO is disputed. While a part of the bioinformatics community considers the GO evaluation irreplaceable, another part points out its weakness, since some GO annotations may be subject to experimental bias or come from not very reliable sources^61^. Unfortunately, at the moment our research community does not have any better computational alternative and I can only notice that further efforts are needed in future studies to develop more accurate methods for evaluation.

However, to compensate for this drawback, I modified the classical GO-based evaluation framework used in the past studies following a more conservative and stringent strategy. It was clearly demonstrated^62^ that comparing the three GO categories, similarities in BP and CC annotations are stronger indicators for protein interaction than similarity in MF annotation. In fact, from a proteomic standpoint, it might be considered arguable to include the MF category when evaluating protein interactions. While it makes sense that two proteins working in the same biological process (or being in the same cellular component) interact, expecting that two proteins with the same molecular function (for example two enzymes) interact, is weak-posed. For this reason, here I propose a more sophisticated and stricter GO-based evaluation framework where the precision is measured: i) excluding MF from the evaluation; ii) considering one unique precision curve estimation, where a protein interaction is considered ‘true’ if it has a Wang similarity value above 0.5 in both BP and CC category at the same time. In practice, I consider as reference a new GO similarity adjacency matrix where each value is computed as the minimum between the respective values in the BP and CC adjacency matrix. It means that in this new evaluation framework an interaction is considered reliable only if its proteins share relevant similarities in terms of biological process and cellular component simultaneously.

In addition, I created a test where three different human networks were considered, one of which was a high-quality and structurally resolved network (the network 7). This was designed to discuss the behaviour of the proposed GO-based evaluation framework. If the new evaluation framework is sufficiently trustworthy, despite the fact that it is very conservative (and thus it strongly penalizes the performance evaluation of the algorithms) we should observe that all the algorithms (even the ones that generally do not perform well) offer a higher performance on this structurally resolved network than in the other networks.

The final GO adjacency matrices (one for each network), which were used to evaluate the algorithm precision, are available at the web-link provided in the Declaration section at the bottom of the article.

## Results and Discussion

The first notable result is that C1* is the best among the proposed algorithms considering their mean performance (Fig. 4D) across all the first 9 networks that are of size (less than 8000 nodes) significantly smaller than the network 10 (around 13000 nodes) of Menche *et al*.^2^. I will discuss separately, at the end of this section, the reliability performance on the network 10 to confirm that the findings attained on networks of smaller size are valid also in this network which represents one of the largest currently available for human interactome^2^.

The mean performance of C1* is significantly better (Mann-Whitney test applied) than the mean performance of the other methods both for AUP (Fig. 5A) and sAUR (Fig. 5B). For instance, Fig. 2 displays the precision curves of the three best algorithms (one for each method category) in network 1, and it is evident that C1* clearly outperforms the others. To provide a simple and clear visualization of the algorithms’ performance, in the next figures I report the plots of the sAUR vs AUP values attained by the different algorithms in the considered networks. C1* offered the best performance in 6/9 networks (Figs 3 and 4) and scored in the first positions in the remaining three (Figs 3 and 4). All the other indices fluctuate in different positions along the evaluations in the diverse networks, and there is not one algorithm that scores always second in general. This is a relevant confirmation of the robustness of the performance attained by C1*. We can interpret this promising result as an indication that C1* might be an important component of an unknown and generalised model useful also to evaluate the reliability of a protein interaction. According to C1* a protein interaction has higher reliability: if the common neighbour proteins (of the seed proteins, Fig. 1) are isolated in a complex (local community) and have low tendency to interact with other external proteins (this message is formally contained in C1 that is the numerator of the C1* mathematical formula); and if the seed proteins also have low propensity to interact with other proteins external to the local community (message contained in the denominator, Fig. 1). Interestingly, I decided to design the C1* formula with exponent two at the denominator, because the operation of squaring a binomial introduces a cross-term $(2\cdot nLC{L}_{x}\cdot \,nLC{L}_{y})$ that particularly penalizes interactions between seed nodes that are both enriched for nLCL. Thus, this cross-term particularly penalizes interactions between proteins that have both high propensity to interact with other proteins external to the local community. Figure 5E,F shows the mean performance of C1* across all the networks for different denominator exponents ranging from 1 to 7. Exponent 2 is the best solution, in fact C1* performance diminishes for exponents larger than 2. This is reasonable because larger exponents introduce additional cross-terms that become predominant in the formula causing a wrong estimation.

The second important result is that all the normalised indices (marked with a circle symbol in Figs 3 and 4) performed better than the non-normalised ones (marked with a triangle symbol in Figs 3 and 4). This difference is statistically significant (Mann-Whitney test applied) across all the networks (Fig. 5C,D). Therefore, the fact that seed proteins have a low propensity to interact with other proteins external to the local community (message contained in the denominator of the normalised algorithms) might be in general an important rule for assessing reliability in PPINs. This explains why PA is always the worst algorithm in any evaluation (Figs 3 and 4) and the cross-term introduced by the exponent 2 in the C1* formula is so effective.

CN is an index that performs very well in social networks (because it is reasonable that the more people you know in common with another person, the higher is the likelihood that you interact with him\her), but here it does not perform efficiently. This suggests that the rule “the more proteins are in common between two proteins, the higher is the likelihood that they interact” might play a secondary part and is not of primary importance for assessing link reliability in PPINs. On the contrary, C1 is a correction of CN where, as discussed above, “the more the common neighbour proteins are isolated in a complex (local community), the higher is the reliability of the seed proteins’ interaction”. This rule is promising because C1 is the best ranked non-normalised index in 8/9 networks (Figs 3 and 4) and scored second (of the non-normalised indices) in the remaining one (Fig. 4A). C1 is also the best non-normalised algorithm for mean performance across all the networks (Fig. 4D). The fact that the C1 rule is performing better than CN has also mere physical motivations due to the molecular scale of the system and the respective driven forces behind the network formation. The morphological and spatial constrains of the operational units assembled in the local communities play an important role for tuning the specific LCP architecture of the network. Here, each protein molecule has a specific 3D steric effect and a selective ability to bind other proteins that are determined by the physical laws respectively at the atomic and molecular scale of the system. Hence, it is expected that the mere CN rule fails, because of a limitation intrinsic to the physical characteristics of the operational unit (the protein) assembled in the network.

The algorithms based on the maximization of the iLCL (C2, C2*, LCL, CAR, CAA, CRA, CJC, CPA) perform in general slightly better than CN (Figs 3 and 4), a fact evident in Fig. 4D where average performances are reported. This suggests that the rule “the more interactions occur between the common neighbour proteins, the higher is the reliability of the seed proteins’ interaction” appears of moderate relevance and certainly less important than C1’s rule, which consists in the minimization of the eLCL (see Fig. 1 and section 2 for details). Interestingly, also in this case the normalization produced a favourable effect. In fact, C2* performed always better than C2: a further confirmation that the low propensity of the seed node proteins to interact with proteins external to the local community might be a relevant factor for link reliability in PPINs.

**Figure 7:**
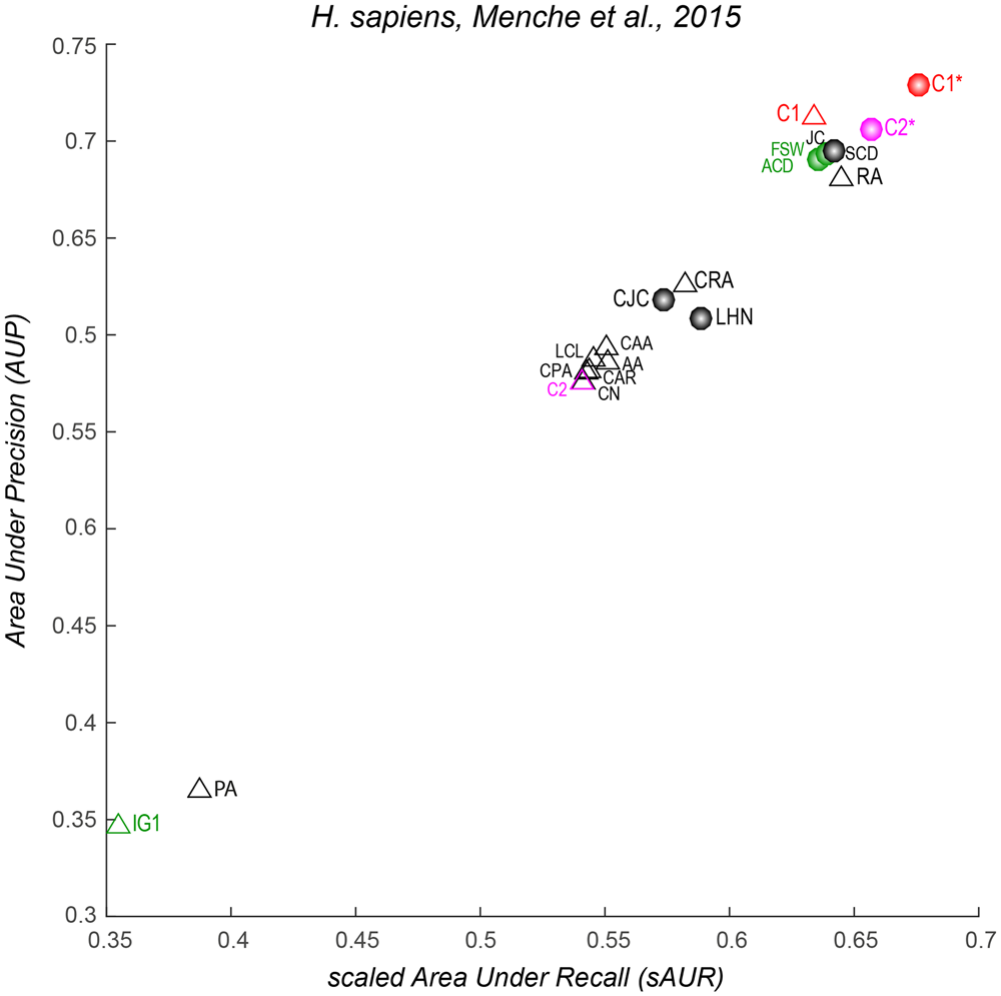
Performance evaluation in network 10. Different colours are used to distinguish: the classical network science reliability models (black); the traditional network biology models (green); the two new local-community models based on the rule C1 and its normalized version C1* (red); the two new local-community models based on the rule C2 and its normalized version C2* (magenta). The triangle symbol is adopted to mark the non-normalised algorithms.

The third key result of the study is that considering the network 10 - which is one of the largest and completed blueprints of the human interactome^2^ - C1 and C1* confirm to be the most precise methods in assessing link reliability (Fig. 7). This finding stresses again the importance to minimize the links external to the local-community in order to correctly assess the reliability of a protein interaction, and might play an important part also to build mechanistic models based on local-community topology for prediction of protein interactions. I will dedicate a separated study to the investigation of this subject.

It is now the turn to discuss the results obtained using the new GO-based evaluation framework. The network 7 is a high quality structurally resolved network, thus we expect that in this network all the algorithms should perform significantly better than in the other networks, and should also give similar performances with a reduced AUP and sAUR variability. In quantitative terms, it means that in network 7 we expect to observe a higher mean algorithm performance with a standard error lower than in the other networks. The plots provided in Fig. 6A,C show that the expected behaviour of the mean and standard error is confirmed by the data; and the coefficient of variation reported in Fig. 6B,D confirms that the extent of performance variability (in relation to mean performance) of all the algorithms in network 7 is significantly lower than in the other networks. Besides to verify that the AUP values obtained by the algorithms in network 7 are statistically different from the performance values obtained in the other networks, I created two groups. In the first group I put the AUP values obtained by the reliability algorithms applied in network 7, and in the second group I put the AUP values obtained by the same algorithms in the other networks. The Mann-Whitney test applied to compare the two groups produced a highly significant statistical difference (p-value < 0.001), suggesting that the proposed GO-evaluation framework is able to emphasize a significant improvement of performance of all the reliability indices in a network that is high quality and structurally resolved. The comparison was repeated also for the sAUR values providing the same significant conclusion. These results considered all together could be expected only if the evaluation framework is well posed. I agree that GO annotations may be subject to experimental bias or come from not very reliable sources. Yet, the framework here proposed, using an integrated approach that combines together the BP and CC categories (to get a more conservative evaluation), provides an encouraging new alternative to deal with this drawback. In fact, considering Fig. 4D (mean performance across the first 9 relatively small size networks) and the Fig. 7 (performance on the large size network 10), we can notice that there is a reasonable matching between the most reliable interactions (according to topological reliability assessed by C1*) and the most reliable according to their biological annotations (considering our new GO-based evaluation framework). This is evident because the AUP values for C1* in Fig. 4D are around 0.6 and in Fig. 7 are around 0.7, which indicates a fair agreement between topological and GO-based scoring. Since I propose to combine the semantic similarities of biological process and cellular component GO-annotations, in a new measure of biological relevance for PPIs, it might be argued that this strategy is too restrictive in comparison to considering the two sematic similarities separately. In order to address this doubt and to clarify the extent to which this can represent a problem, I computed the area under the precision-recall curve (AUPR) of each method in each network considering separately (Suppl. Table 1 to Suppl. Table 10): biological process (BP), cellular component (CC), molecular function (MF) and the new strategy I propose that is the intersection of BP and CC, therefore it is indicated with I(B,C). In order to offer a general comparison of the 4 different evaluation strategies, I summarized in Table 1 what is the best method (it means ranked number 1 for AUPR) in each network according to the four different evaluation paradigms. The result is that both BP and CC have a congruent evaluation trend with I(B,C) that dispels the suspicion that I(B,C) is too restrictive. On the other hand, it appears evident that, as conjectured in the Methods, MF is not a proper measure to point out the best reliability method because it does not bring to any clear conclusion across the networks. In addition, we can gather that, also according to this evaluation based on AUPR, C1* seems the best mechanistic model across our experiments to assess link reliability in protein interactomes.

**Table 1:** Top method in each network according to AUPR performance measure considering different gene-ontology-based (GO-based) evaluations.

Network	AUPR (BP)	AUPR (CC)	AUPR (MF)	AUPR I(B,C)	Ranking	
					Method	Count
Yeast (Ben Hur *et al*.^27^	C1*	C1*	FSW	C1*		
Yeast (Chen *et al*.^22^	C1*	C1*	FSW	C1*	C1*	**18/40**
Yeast (Das *et al*.^28^	C1*	FSW	C1*	C1*	FSW	11/40
Plant (AIMC 2011)	FSW	FSW	ACD	FSW	RA	4/40
Worm (BioGRID 2012)	FSW	FSW	CPA	ACD	CPA	2/40
Fly (BioGRID 2012)	C1*	C1*	C1*	C1*	ACD	2/40
Human (Wang *et al*.^20^	RA	C1*	PA	RA	C1	1/40
Human (Das *et al*.^28^	C1*	C1	RA	C1*	C2*	1/40
Human (Rolland *et al*.^31^	FSW	FSW	C2*	FSW	PA	1/40
Human (Menche *et al*.^2^	C1*	RA	CPA	C1*		
Total occurrence	C1* (6/10)	C1* (4/10)	—	C1* (6/10)		

From the left, the first column reports the name of the network considered. Second to fifth columns report the best method according to the following GO-based evaluations: biological process (BP), cellular component (CC), molecular function (MF) and the new strategy I propose that is the intersection of BP and CC, therefore it is indicated with I(B,C). On the right part of the table, it is reported the ranking of the methods. This ranking is obtained by counting for each method the number of times it arrives first across all the four different evaluations considering all the networks.

Hereafter, we will take care to discuss the issues and possible solutions associated to the network incompleteness. PPIs are known to be incomplete and thus there might be a research bias not only in terms of GO but also in terms of topological biases. In fact, well-studied proteins might have more connections merely by the fact that they are well studied, while orphaned proteins might be disproportionately ‘underconnected’ by research bias. The topological incompleteness might influence the performance of these methods that rely only on network topology. Future studies might consider computational experiments aimed to quantitatively investigate the extent to which the performance of these methods is modified by introducing controlled network topology perturbations. A first question could be to assess whether some algorithms are specifically susceptible to edge removal quantifying a possible performance drop. For instance, the performance of methods for link reliability or prediction can be evaluated with networks were 10% or 20% of the edges are removed uniformly at random (a procedure known as link sparsification). However, it should be remarked that this procedure, if not correctly applied, can represent a bias itself, in fact removing percentage of links larger than 20% can be misleading because the removal procedure does not respect the intrinsic rules of growth of the interactome, and therefore can introduce a bias by its own in the organization of the network topology. Nevertheless, in one of my previous studies^8^ I showed that local-community-based models (such as CAR) display a good performance robustness in protein interaction prediction when PPINs are subject to progressive link sparsification. On the other hand, other important questions related to network topology perturbation experiments could be to investigate whether some algorithms are particularly susceptible to edge rewiring (for instance rewiring under preservation of degree distribution) or random addition of false-positive links. An interesting idea could be to test how the methods’ performance is modified when a certain percentage of random edges which should possess low link reliability is added to the original topology. For instance, add 10% random edges with low link reliability according to GO-annotation and evaluate how many of those pop up among the 10% edges predicted to be most reliable or least reliable. This would give a further indication for the network de-noising capabilities of the different methods. Furthermore, similar experiments could be repeated using generative models which resemble the topological features (average degree, average clustering, etc.) of real protein interactomes^63^ and could represent a ground-truth for benchmarking how different methods behave in presence of topological perturbation. On this regard, a first solution could be to adopt the duplication-mutation/divergence model which was specifically created^64^ and further developed^65^ for protein networks, although a recent study spotted that it presents many limitations^66^. Another solution could be to adopt new classes of soft random geometrical graphs, such as the nonuniform-popularity-similarity-optimization model^67,68^, that allow a fine tuning of many network features such as clustering, small-wordness, node heterogeneity, rich-clubness and community structure. Yet, the limitation of using generative models for generation of synthetic protein interactome is that nobody know the authentic generative model behind these networks, therefore it is better to fairly compare the performance using diverse types and classes of generative models, in the hope to converge to results that resemble the ones observed using real networks. To conclude this paragraph, it is clear that future studies should put additional effort especially to investigate and develop gold standard methods for evaluation of algorithms which perform link reliability and prediction in PPINs.

To summarize, this study is aimed to investigate local-structure similarity-based models, and to disclose and understand some of the constitutive topological rules that emerge from the mechanistic forces behind PPINs’ self-organization. The advantage of these local mechanistic models - in respect to global models such as machine learning methods - is that they are based on an explicit and interpretable rule of organization. Whereas, unlikely, machine learning methods build (learn) an implicit model that in general cannot be disclosed in an interpretable rule of organization. However, many local models have the limitation that they cannot assign reliability to interactions that do not present common neighbours, a drawback not affecting global methods. A previous study showed that the local-community-paradigm models can significantly outperform some methods based on machine learning dimension reduction in link prediction of PPNIs^8,36^ and drug-target interactions^37^, but new advanced global methods have been recently published^3,4,7,9,69^. Future studies on link reliability, topological denoising and confidence scoring should compare the novel local models with the state-of-the-art global methods, with the aim to unveil and analyse what are the common set of links correctly scored by both the classes of predictors and what are, instead, the set of links specifically and correctly scored by each single class or method. A careful examination should help to clarify what are the strategies to merge the predictions of heterogeneous methods in a unique tool such as IntScore^4^ that, with the rise of network medicine, will become increasingly useful for reliable network-based computational analysis in precision and systems medicine^2,11,70,71^.

## Electronic supplementary material

Table S1

## Data Availability

The data supporting this article are available at: https://sites.google.com/site/carlovittoriocannistraci/home.
