# Supplementary material for: Modelling Self-Organization in Complex Networks Via a Brain-Inspired Network Automata Theory Improves Link Reliability in Protein Interactomes
Source: Sci Rep. 2018 Oct 25;8:15760. doi: 10.1038/s41598-018-33576-8 (PMC6202355; doi:10.1038/s41598-018-33576-8)
Supplement: Supplementary file 1 — Table S1 [file 41598_2018_33576_MOESM1_ESM.pdf]

Supplementary Information

MODELLING LINK RELIABILITY IN COMPLEX NETWORKS VIA A BRAIN-INSPIRED NETWORK AUTOMATA SELF-ORGANIZATION THEORY IMPROVES TOPOLOGICAL DENOISING IN PROTEIN INTERACTOMES

Carlo Vittorio Cannistraci[1,2,*]

[1]Biomedical Cybernetics Group, Biotechnology Center (BIOTEC), Center for Molecular and Cellular Bioengineering (CMCB), Center for Systems Biology Dresden (CSBD), Department of Physics, Technische Universität Dresden, Tatzberg 47/49, 01307 Dresden, Germany

[2]Brain bio-inspired computing (BBC) lab, IRCCS Centro Neurolesi "Bonino Pulejo", Messina, Italy

*Corresponding author: kalokagathos.agon@gmail.com

Network 1	AUPR_BP	AUPR_CC	AUPR_MF	AUPR_In(BP,CC)
C1*	**0.347**	**0.510**	0.217	**0.336**
LHN	0.327	0.506	0.208	0.304
JC	0.316	0.498	0.192	0.284
SCD	0.316	0.498	0.192	0.284
ACD	0.310	0.496	0.186	0.277
C2*	0.276	0.484	0.165	0.251
FSW	0.282	0.496	**0.222**	0.243
C1	0.255	0.483	0.175	0.213
RA	0.248	0.482	0.172	0.206
CJC	0.220	0.463	0.153	0.176
CRA	0.218	0.461	0.155	0.174
AA	0.221	0.474	0.162	0.167
CAA	0.207	0.458	0.152	0.162
CAR	0.201	0.457	0.151	0.152
LCL	0.202	0.457	0.151	0.149
C2	0.201	0.457	0.151	0.147
CN	0.200	0.469	0.153	0.143
IG1	0.164	0.457	0.171	0.127
CPA	0.149	0.427	0.119	0.112
PA	0.079	0.413	0.102	0.050

Suppl. Table 1. AUPR values of the different methods, which are on the rows, in Network 1. The results are provided for four different gene ontology based evaluations, which are on the columns. In bold the maximum performance for each column. The methods are ranked in decreasing order of performance (according to the fourth column) starting from the best one, which is the first at the top of the table.

network 2	AUPR_BP	AUPR_CC	AUPR_MF	AUPR_In(BP,CC)
C1*	**0.440**	**0.587**	0.295	**0.429**
JC	0.416	0.576	0.273	0.394
SCD	0.416	0.576	0.273	0.394
LHN	0.416	0.580	0.277	0.392
ACD	0.409	0.575	0.268	0.386
FSW	0.414	0.571	**0.304**	0.382
C2*	0.357	0.551	0.240	0.343
C1	0.363	0.562	0.250	0.333
RA	0.343	0.559	0.235	0.308
CRA	0.290	0.521	0.207	0.252
CJC	0.286	0.518	0.202	0.245
AA	0.285	0.539	0.204	0.236
CAA	0.257	0.509	0.190	0.214
LCL	0.243	0.504	0.183	0.197
CAR	0.242	0.504	0.181	0.196
C2	0.242	0.505	0.181	0.196
CN	0.247	0.527	0.183	0.193
IG1	0.203	0.506	0.190	0.155
CPA	0.182	0.480	0.147	0.147
PA	0.094	0.462	0.105	0.065

Suppl. Table 2. AUPR values of the different methods, which are on the rows, in Network 2. The results are provided for four different gene ontology based evaluations, which are on the columns. In bold the maximum performance for each column. The methods are ranked in decreasing order of performance (according to the fourth column) starting from the best one, which is the first at the top of the table.

Network 3	AUPR_BP	AUPR_CC	AUPR_MF	AUPR_In(BP,CC)
C1*	**0.421**	0.662	**0.252**	**0.437**
LHN	0.398	0.656	0.240	0.405
FSW	0.405	**0.670**	0.252	0.405
JC	0.320	0.657	0.223	0.325
SCD	0.320	0.657	0.223	0.325
ACD	0.302	0.655	0.216	0.305
C1	0.295	0.654	0.222	0.298
C2*	0.260	0.620	0.196	0.263
RA	0.262	0.652	0.210	0.262
AA	0.176	0.643	0.173	0.166
CRA	0.166	0.613	0.165	0.160
CJC	0.147	0.610	0.154	0.140
CAA	0.140	0.608	0.152	0.132
IG1	0.146	0.610	0.141	0.123
LCL	0.129	0.605	0.146	0.121
C2	0.128	0.606	0.146	0.120
CAR	0.128	0.605	0.145	0.119
CN	0.130	0.632	0.150	0.118
CPA	0.049	0.568	0.104	0.044
PA	0.023	0.551	0.089	0.018

Suppl. Table 3. AUPR values of the different methods, which are on the rows, in Network 3. The results are provided for four different gene ontology based evaluations, which are on the columns. In bold the maximum performance for each column. The methods are ranked in decreasing order of performance (according to the fourth column) starting from the best one, which is the first at the top of the table.

Network 4	AUPR_BP	AUPR_CC	AUPR_MF	AUPR_In(BP,CC)
FSW	**0.307**	**0.539**	0.564	**0.331**
C1*	0.280	0.515	0.564	0.325
JC	0.275	0.514	0.566	0.319
SCD	0.275	0.514	0.566	0.319
ACD	0.273	0.514	**0.567**	0.317
LHN	0.273	0.512	0.552	0.298
C1	0.260	0.511	0.559	0.287
RA	0.253	0.511	0.557	0.279
AA	0.245	0.508	0.556	0.258
C2*	0.241	0.482	0.530	0.255
CJC	0.224	0.481	0.529	0.226
CRA	0.223	0.480	0.529	0.226
CN	0.227	0.498	0.547	0.220
CAA	0.219	0.480	0.528	0.219
LCL	0.215	0.478	0.527	0.212
C2	0.213	0.479	0.526	0.211
CAR	0.214	0.478	0.526	0.210
IG1	0.209	0.447	0.486	0.159
CPA	0.138	0.379	0.453	0.091
PA	0.078	0.339	0.390	0.028

Suppl. Table 4. AUPR values of the different methods, which are on the rows, in Network 4. The results are provided for four different gene ontology based evaluations, which are on the columns. In bold the maximum performance for each column. The methods are ranked in decreasing order of performance (according to the fourth column) starting from the best one, which is the first at the top of the table.

Network 5	AUPR_BP	AUPR_CC	AUPR_MF	AUPR_In(BP,CC)
ACD	0.317	0.481	0.742	**0.236**
C1*	0.312	0.484	0.730	0.231
JC	0.317	0.480	0.738	0.231
SCD	0.317	0.480	0.738	0.231
LHN	0.314	0.478	0.727	0.227
FSW	**0.334**	**0.515**	0.665	0.225
C1	0.300	0.471	0.743	0.205
RA	0.298	0.471	0.744	0.205
AA	0.299	0.465	0.748	0.200
CN	0.297	0.446	0.754	0.182
C2*	0.319	0.448	0.717	0.175
CJC	0.318	0.442	0.717	0.170
CRA	0.316	0.442	0.717	0.161
CAA	0.316	0.438	0.717	0.159
LCL	0.316	0.436	0.717	0.157
CAR	0.314	0.437	0.717	0.154
C2	0.314	0.437	0.717	0.154
IG1	0.309	0.498	0.665	0.110
CPA	0.215	0.347	**0.768**	0.077
PA	0.195	0.339	0.766	0.059

Suppl. Table 5. AUPR values of the different methods, which are on the rows, in Network 5. The results are provided for four different gene ontology based evaluations, which are on the columns. In bold the maximum performance for each column. The methods are ranked in decreasing order of performance (according to the fourth column) starting from the best one, which is the first at the top of the table.

Network 6	AUPR_BP	AUPR_CC	AUPR_MF	AUPR_In(BP,CC)
C1*	**0.256**	**0.525**	**0.261**	**0.264**
LHN	0.231	0.519	0.216	0.257
C2*	0.222	0.511	0.232	0.227
FSW	0.209	0.509	0.225	0.207
JC	0.202	0.523	0.221	0.202
SCD	0.202	0.523	0.221	0.202
ACD	0.196	0.522	0.215	0.195
C1	0.183	0.516	0.217	0.185
RA	0.138	0.514	0.159	0.149
CRA	0.107	0.500	0.135	0.109
CJC	0.102	0.499	0.124	0.108
AA	0.087	0.508	0.108	0.093
CAA	0.085	0.495	0.105	0.091
LCL	0.080	0.493	0.098	0.085
CAR	0.078	0.493	0.096	0.083
CPA	0.077	0.487	0.096	0.083
C2	0.076	0.493	0.093	0.083
CN	0.076	0.504	0.093	0.082
IG1	0.055	0.393	0.063	0.061
PA	0.021	0.429	0.054	0.015

Suppl. Table 6. AUPR values of the different methods, which are on the rows, in Network 6. The results are provided for four different gene ontology based evaluations, which are on the columns. In bold the maximum performance for each column. The methods are ranked in decreasing order of performance (according to the fourth column) starting from the best one, which is the first at the top of the table.

Network 7	AUPR_BP	AUPR_CC	AUPR_MF	AUPR_In(BP,CC)
RA	**0.413**	0.749	0.816	**0.376**
C1	0.412	0.749	0.812	0.374
C1*	0.397	**0.753**	0.813	0.366
ACD	0.398	0.752	0.810	0.365
JC	0.397	0.751	0.810	0.365
SCD	0.397	0.751	0.810	0.365
AA	0.407	0.744	0.815	0.364
CN	0.392	0.734	0.813	0.343
FSW	0.369	0.749	0.786	0.343
LHN	0.373	0.743	0.810	0.334
C2*	0.372	0.741	0.804	0.323
CRA	0.376	0.739	0.802	0.322
CJC	0.376	0.739	0.803	0.321
CAA	0.375	0.738	0.802	0.318
CAR	0.376	0.736	0.805	0.318
C2	0.375	0.735	0.805	0.316
LCL	0.373	0.735	0.804	0.314
CPA	0.356	0.712	0.811	0.282
IG1	0.330	0.723	0.785	0.279
PA	0.327	0.701	**0.823**	0.258

Suppl. Table 7. AUPR values of the different methods, which are on the rows, in Network 7. The results are provided for four different gene ontology based evaluations, which are on the columns. In bold the maximum performance for each column. The methods are ranked in decreasing order of performance (according to the fourth column) starting from the best one, which is the first at the top of the table.

Network 8	AUPR_BP	AUPR_CC	AUPR_MF	AUPR_In(BP,CC)
C1*	**0.282**	0.661	0.614	**0.293**
JC	0.272	0.658	0.613	0.283
SCD	0.272	0.658	0.613	0.283
ACD	0.270	0.659	0.613	0.281
C1	0.267	**0.663**	0.621	0.277
RA	0.264	0.662	**0.622**	0.274
FSW	0.274	0.622	0.550	0.268
AA	0.246	0.658	0.621	0.250
LHN	0.248	0.651	0.604	0.246
C2*	0.216	0.651	0.584	0.221
CRA	0.214	0.652	0.588	0.220
CN	0.218	0.653	0.619	0.217
CJC	0.211	0.650	0.586	0.216
CAA	0.208	0.650	0.587	0.212
LCL	0.204	0.649	0.587	0.205
CAR	0.204	0.648	0.588	0.204
C2	0.204	0.648	0.588	0.204
CPA	0.129	0.631	0.613	0.128
IG1	0.143	0.610	0.552	0.126
PA	0.079	0.624	0.615	0.072

Suppl. Table 8. AUPR values of the different methods, which are on the rows, in Network 8. The results are provided for four different gene ontology based evaluations, which are on the columns. In bold the maximum performance for each column. The methods are ranked in decreasing order of performance (according to the fourth column) starting from the best one, which is the first at the top of the table.

Network 9	AUPR_BP	AUPR_CC	AUPR_MF	AUPR_In(BP,CC)
FSW	**0.111**	**0.465**	0.509	**0.113**
C1*	0.076	0.434	0.520	0.078
JC	0.075	0.413	0.523	0.075
SCD	0.075	0.413	0.523	0.075
LHN	0.065	0.426	0.503	0.068
ACD	0.070	0.408	0.528	0.064
C2*	0.059	0.415	**0.537**	0.056
IG1	0.046	0.443	0.497	0.050
C1	0.055	0.419	0.531	0.050
RA	0.052	0.413	0.533	0.042
CRA	0.042	0.403	0.531	0.032
CJC	0.030	0.397	0.537	0.020
AA	0.028	0.386	0.531	0.016
CAA	0.024	0.383	0.526	0.013
LCL	0.017	0.379	0.526	0.007
CN	0.016	0.378	0.530	0.007
CAR	0.017	0.391	0.533	0.006
C2	0.016	0.391	0.533	0.006
CPA	0.013	0.339	0.530	0.004
PA	0.010	0.339	0.521	0.003

Suppl. Table 9. AUPR values of the different methods, which are on the rows, in Network 9. The results are provided for four different gene ontology based evaluations, which are on the columns. In bold the maximum performance for each column. The methods are ranked in decreasing order of performance (according to the fourth column) starting from the best one, which is the first at the top of the table.

Network 10	AUPR_BP	AUPR_CC	AUPR_MF	AUPR_In(BP,CC)
C1*	**0.435**	0.769	0.637	**0.441**
C2*	0.410	0.767	0.623	0.417
C1	0.401	0.775	0.662	0.407
JC	0.390	0.773	0.649	0.399
SCD	0.390	0.773	0.649	0.399
FSW	0.386	0.760	0.638	0.397
ACD	0.381	0.773	0.650	0.391
RA	0.382	**0.777**	0.666	0.391
LHN	0.351	0.748	0.608	0.339
CRA	0.331	0.769	0.652	0.333
CJC	0.325	0.768	0.649	0.326
CAA	0.308	0.767	0.654	0.307
AA	0.304	0.772	0.674	0.306
LCL	0.303	0.767	0.654	0.303
CAR	0.300	0.767	0.655	0.299
CPA	0.299	0.762	**0.675**	0.298
CN	0.296	0.772	0.671	0.297
C2	0.297	0.767	0.656	0.296
PA	0.147	0.741	0.672	0.142
IG1	0.139	0.690	0.572	0.123

Suppl. Table 10. AUPR values of the different methods, which are on the rows, in Network 10. The results are provided for four different gene ontology based evaluations, which are on the columns. In bold the maximum performance for each column. The methods are ranked in decreasing order of performance (according to the fourth column) starting from the best one, which is the first at the top of the table.
